# Blast Waves Cause Immune System Dysfunction and Transient Bone Marrow Failure in a Mouse Model

**DOI:** 10.3389/fbioe.2022.821169

**Published:** 2022-03-22

**Authors:** Elke S. Bergmann-Leitner, Alexander G. Bobrov, Jessica S. Bolton, Michael D. Rouse, Lanier Heyburn, Radmila Pavlovic, Brittany I. Garry, Yonas Alamneh, Joseph Long, Brett Swierczewski, Stuart Tyner, Derese Getnet, Venkatasivasai S. Sajja, Vlado Antonic

**Affiliations:** ^1^ Biologics Research and Development, Walter Reed Army Institute of Research, Silver Spring, MD, United States; ^2^ Wound Infections Department, Walter Reed Army Institute of Research, Silver Spring, MD, United States; ^3^ Wound Infections Department, Naval Research Medical Center, Silver Spring, MD, United States; ^4^ Henry M. Jackson Foundation, Rockville, MD, United States; ^5^ Blast Induced Neurotrauma Branch, Walter Reed Army Institute of Research, Silver Spring, MD, United States; ^6^ Bacterial Disease Branch, Walter Reed Army Institute of Research, Silver Spring, MD, United States; ^7^ Military Infectious Diseases Research Program, Frederick, MD, United States

**Keywords:** blast wave, immune response, long term effects, bone marrow, spleen, hematopoietic stem cells, progenitor cells, cytokine

## Abstract

Explosive devices, either conventional or improvised, are common sources of injuries during combat, civil unrest, and terror attacks, resulting in trauma from exposure to blast. A blast wave (BW), a near-instantaneous rise in pressure followed by a negative pressure, propagates through the body in milliseconds and can affect physiology for days/months after exposure. Epidemiological data show that blast-related casualties result in significantly higher susceptibility to wound infections, suggesting long-lasting immune modulatory effects from blast exposure. The mechanisms involved in BW-induced immune changes are poorly understood. We evaluated the effects of BW on the immune system using an established murine model. Animals were exposed to BWs (using an Advanced Blast Simulator), followed by longitudinally sampling for 14 days. Blood, bone marrow, and spleen were analyzed for changes in the 1) complete blood count (CBC), and 2) composition of bone marrow cells (BMC) and splenocytes, and 3) concentrations of systemic cytokines/chemokines. Our data demonstrate that BW results in transient bone marrow failure and long-term changes in the frequency and profile of progenitor cell populations. Viability progressively decreased in hematopoietic stem cells and pluripotent progenitor cells. Significant decrease of CD4^+^ T cells in the spleen indicates reduced functionality of adaptive immune system. Dynamic changes in the concentrations of several cytokines and chemokines such as IL-1α and IL-17 occurred potentially contributing to dysregulation of immune response after trauma. This work lays the foundation for identifying the potential mechanisms behind BW’s immunosuppressive effects to inform the recognition of this compromised status is crucial for the development of therapeutic interventions for infections to reduce recovery time of wounded patients injured by explosive devices.

## Introduction

Wound infections are an enduring threat to Warfighters, as well as civilians caught in war, public unrest, and terror attacks. Injuries to extremities accounted for 65% of the wounds to soldiers in Operation Iraqi Freedom and Operation Enduring Freedom (Murray, 2008). The majority of these injuries were sustained from explosive devices (e.g., improvised explosive device (IED), mortars, and grenades) (Dougherty et al., 2009). Shrapnel wounds from the explosions are often compounded and further complicated by environmental debris that contains various microbes which, when embedded in a penetrating wound, cause infection. Explosion-related injuries are complex in nature, characterized by large tissue defects and deep embedding of bacterial contaminants within the soft tissues (Melvin et al., 2010; Rozen and Lerner, 2011). However, the increase in initial survival and the penetrating nature of injuries has given rise to the development of delayed pathologies and morbidities. Wound infections occurred in approximately 25–30% of wounded soldiers and presented a major source of morbidity and mortality in injured service members (Sheppard et al., 2010; Hospenthal et al., 2011; Hospenthal and Murray, 2011). Incidence of infections and sepsis is expected to be an even greater concern in future joint domain operations threat scenarios (Army, 2018). In these threat scenarios, deployment of IEDs and conventional explosives are and will continue to be the most common cause of injury, requiring prolonged field care. With limited access to medevac, robust rear element medical care infrastructures are anticipated to be the norm in future threat scenarios for military casualties (Army, 2018). Limited data are available on the effect of blast exposure on wound healing/wound infection, and our lack of understanding of blast-related wounds/wound infections likely results in sub optimal care for blast-related injuries.

Explosion-related BW exposure lasts in the order of milliseconds. However, its effects on the body can last for long after exposure and involve interlinked mechanisms of systemic, local and cerebral responses. It has been well documented that BW exposure leads to air embolism (Kirkman and Watts, 2011; van H. Mason et al., 1971), activation of the autonomic nervous system (ANS) (Cernak et al., 1996; Tümer et al., 2013), compromised blood flow throughout the body (Health, 2014), blood brain barrier disruption (Kuriakose et al., 2018), systemic inflammation (Surbatovic et al., 2007; Cernak, 2010), along with damage to the lungs (Elsayed and Gorbunov, 2007; Koliatsos et al., 2011; Sajja et al., 2020) and intestines (Cripps and Cooper, 1997; Bala et al., 2008). As the BW propagates through the entire body and all the tissues, it triggers numerous biochemical and physiological changes, such as the release of reactive oxygen species (ROS) and subsequent damage to proteins (Cho et al., 2013), damage associated molecular patterns (DAMPs) released from necrotic cells, general tissue hypoxia due to compromised blood flow, and immunological changes both locally and systemically (Thompson et al., 2019). Several reports have associated blast wounds with a higher likelihood of infection development without providing mechanistic insights (Blyth et al., 2015; Burns et al., 2012; D'Alleyrand et al., 2015; office, 2016). In the study performed by the Trauma Infection disease Outcome Study (TIDOS) program, infection was more than twice as likely to develop in blast-exposed casualties compared with those possessing similar injuries but without concomitant blast exposure (Stewart et al., 2019). The increase in infection susceptibility described in these reports leads us to hypothesize that blast exposure results in the long-term changes in the immune system at a phenotypic level at the bone marrow. Moreover, a recently published review on the tiered consequences of blast injuries acknowledged the long-term impact on both innate and adaptive immunity (Thompson et al., 2019). Despite the significant challenges they pose to military and civilian medicine, the deleterious effects of BW on the immune system is a surprisingly understudied area.

The main objective of the present study was to identify changes in the immune system caused by BW and take the first steps in understanding potential mechanisms responsible for those observed changes in immune responses. The results of this study strongly imply that BW causes broad-range of changes that resonate throughout the immune system that may contribute to the development and prolongation of wound infections in explosion-related casualties.

## Materials and Methods

### Ethical Consideration

All animal experiments were conducted at Walter Reed Army Institute of Research (an AAALAC International accredited research facility) in accordance with the Animal Welfare Act and other federal statutes and regulations relating to animals and experiments involving animals. The animal use protocols were approved by the Institutional Animal Care and Use Committee at the Walter Reed Army Institute of Research [Assurance number D16-00596 (A4117-01)] and adhered to principles stated in the Guide for the Care and Use of Laboratory Animals (NRC Publication, 2011 edition) using an Institutional Animal Care and Use Committee approved protocol (18-BRD-16S).

### Animals and Housing

For this study 8–12 week old male BALB/c mice (Jackson Laboratories, USA) were used and housed at 20–22°C (12-h light/dark cycle). Mice were provided food (Prolab IsoPro RMH3000 from LabDiet, St. Louis, MO) and water *ad libitum.*

#### Experimental Groups

Animals were randomly assigned to three experimental groups (n = 6 mice per group and time point) and samples collected in parallel: (Group 1) Sham-treatment. Animals were subjected to isoflurane anesthesia, loaded into the ABS, and underwent recovery procedures as the BW group; (Group 2) BW treatment; (Group 3) Cyclophosphamide (CP) treatment. The impact of BW was assessed by euthanizing cohorts of mice at various time points (Day 0–7, Day 14) to generate longitudinal data sets. CP-treated mice were used as positive control to emulate immunosuppression as it induces neutropenia and enables infection establishment (Jacobs et al., 2014; Thompson et al., 2014; Thompson et al., 2015). Pretreatment with CP results in severe but transient neutropenia which resolves in 3–5 days depending on the dose and route of administration (Huyan et al., 2011). CP-treated positive controls were used for direct comparison to BW-exposed animals to emulate transient changes (3–5 days) similar to the time-course of infections in combat operations. CP was used to serve as end-state neutropenia control to understand the extent of BW effects on circulating blood cells.

#### Blast Wave Exposure

Animals were exposed to BW at 19 psi according to our published procedure, which constitutes non-lethal pressure. Animals were anesthetized using isoflurane (5% isoflurane, 8 min) (Antonic et al., 2020). Thereafter, animals were positioned in side-on position inside the advanced blast simulator (Patent US20130042665A1) and exposed to a single ∼19 psi BW with an impulse of ∼29.87 psi*msec.

### Sample Collection

Cohorts of animals (n = 4–6/group and time point) were euthanized daily on days 1–7 and day 14 after blast exposure. At the time of euthanasia, blood was collected via cardiac puncture and 200 µl aliquots were transferred into purple top EDTA tubes. The remaining blood was used for serum collection. Femurs were dissected and bone marrow cells were isolated according to the published protocol (Liu and Quan, 2015). Spleens were removed and minced through a sterile 70 µm nylon filter (BD Falcon, Franklin Lakes, NJ) to obtain a single cell suspension, washed, and cell pellets resuspended in a cryopreservative medium (90% FBS/10% DMSO) and frozen for later analysis.

### Differential Whole Blood Cell Count

Whole blood was collected by cardiac puncture into EDTA tubes. Complete blood cell count was analyzed using an automated cell counter according to the manufacturer’s instructions (HemaVet950, Drew Scientific, USA). Serum was collected and stored at−80°C until further analysis.

### Inflammatory Mediators

Serum cytokine/chemokine concentrations were assayed using a cytokine premixed 25 panel magnetic immunology assay (Luminex, USA) or a 29-plex u-plex electro-chemiluminescence based multiplex platform (MesoScale Discovery, USA) in accordance with manufacturer’s protocol (Kaba et al., 2018). The factors quantified were: (a) chemokines: IP-10, MIP-1α, MCP-1, MIP-2, MIP-3α, (b) pro-inflammatory factors: IL-1β, IL-6, KC/GRO, IL-22, (c) Th1: IL2, IL-15, IL-12p70, IFN-α, TNF-α, (d) Th2: IL-4, IL-5, IL-31, (e) Th17: IL-23, IL-17A, IL-17C, IL-17E, IL-17F, and (f) regulatory cytokines: IL-9, IL-10, IL-21, IL-27, IL-33.

### Flow Cytometry

#### Phenotyping of Bone Marrow Cells

Characterization of the phenotype and frequency of hematopoietic stem and progenitor cells (HSPCs) from bone marrow was determined through multi-parametric flow cytometry (Technology, 2018). Cryopreserved HSPCs were thawed, washed twice and counted (LUNA-FL cell counter, Logos Biosystems, Annandale, VA) in cRPMI [RPMI 1640 containing 25 mM HEPES, with 1% MEM NEAA, 1% Sodium Pyruvate, 1% Penicillin/Streptomycin mixture, 2 mM l-glutamine (Quality Biology, Gaithersburg, MD), 0.05 mM ß-mercaptoethanol (Thermofisher Scientific, Waltham, MA), and 10% fetal calf serum (Thermofisher Scientific)]. Cells from treated animals were recovered after freezing at the same rate as cells from control animals, indicating that treatment does not alter the viability of cell populations during cryopreservation. After adjusting the cell concentration, cells were washed twice with FACS buffer [1X PBS (Thermofisher scientific), 0.05% sodium azide (Ricca Chemical Company, Arlington, TX), and 1% rat serum (BioVT, Westbury, NY)]. CD16/CD32 (mouse BD Fc block, clone 2.4G2, BD Biosciences, San Jose, CA), was added to the cells at 1 μg/ml in FACS buffer, incubated for 10 min at 4°C. Cells were washed twice with FACS buffer. The panels consisted of:

The mouse LSK phenotyping panel: CD3 (FITC, clone 145-2C11), CD11b (FITC, clone M170), CD45R (FITC, clone RA3-6B2), Gr-1 (FITC, clone RB6-8C5), TER119 (FITC, clone TER119), c-Kit (PE, clone 2B8) and Sca1 (APC, clone E13-161.7).

The mouse LSK/SLAM phenotyping panel: CD3 (FITC, clone 145-2C11), CD11b (FITC, clone M170), CD45R (FITC, clone RA3-6B2), Gr-1 (FITC, clone RB6-8C5), TER119 (FITC, clone TER119), c-Kit (PE, clone 2B8), CD48 (APC, clone HM48-1), CD150 (PECy7, clone TC15-12F12.2) Sca1 (Biotin, clone E13-161.7) and Sca1 (Streptavidin BV 421, clone B316686).

The mouse ESLAM phenotyping panel: CD45 (Alexa Four 488, clone 30-F11), CD48 (APC, clone HM48-1), CD150 (PECy7, clone TC15-12F12.2) and EPCR (PE, clone RMEPCR1560).

All antibodies were obtained from Stemcell Technologies, Cambridge, MA except Sca1 BV421, which was obtained from Biolegend, San Diego, CA. For each panel, the antibodies were added to the cells and incubated for 30 min at room temperature. The cells were washed twice and re-suspended in FACS buffer. Fifteen minutes prior to acquisition, 1 μg/ml of 7-AAD (Thermofisher Scientific) was added to all samples to detect cell viability. The samples were acquired using the Miltenyi Biotec MACSQuant Analyzer 10, in the ImmunoCore facility using the MACSQuantify software 2.6 (Technology, 2018) and analyzed using FlowJo 10.4 software (see Supplementary Figure S1 for gating strategy).

#### Phenotyping of Splenocytes

Cryopreserved splenocytes were thawed, washed with FACS buffer, and cell counts determined as described for bone marrow. CD16/CD32 (mouse BD Fc block, clone 2.4G2, BD Biosciences, San Jose, CA), was added to the cells at 1 μg/ml in FACS buffer, incubated for 10 min at 4°C. Cells were washed twice with FACS buffer. A cocktail of the following antibodies was added to the cells: CD3 (AF, clone 17A2), CD4 (PerCP-Cy5.5, clone RM4-5), CD8 (PE-Cy7, clone 53–6.7). 7-AAD was added 15 min prior to sample acquisition on a MACSQuant Analzyer 10 as described above.

### Statistics

Statistically significant differences between time points were identified by two-way ANOVA after testing for equal variance and normal distribution. Significant differences between discrete time points and controls (sham-treated animals) were determined by Dunnett’s tests (Minitab 18, State College, PA, USA).

## Results

The current study sought to determine the cause for increased susceptibility to infections after BW. The first step was to assess changes in the frequency and composition of cell populations in peripheral blood of mice after receiving a single BW, then monitor the dynamic changes in the spleen and bone marrow of BW-exposed mice. Treatment of the mice with cyclophosphamide (CP), an immunosuppressive drug that produces well-established and reproducible effects on CBC, was used as a positive control.

### Blast Wave Exposure Results in Changes in the Frequency and Composition of Circulating Immune Cells

**FIGURE 1:**
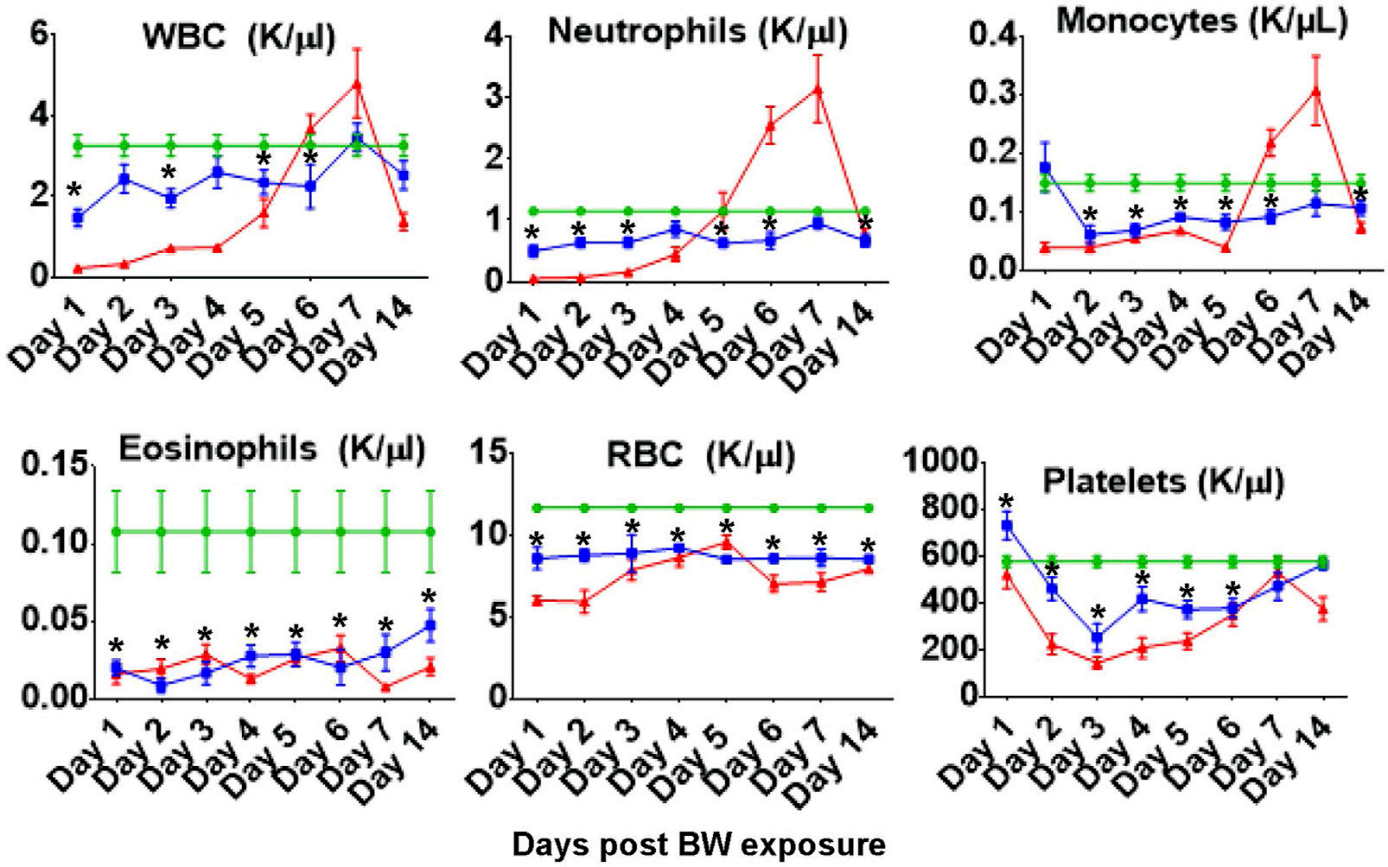
Blast exposure significantly alters number of circulating blood cells throughout the 14 days follow up. Data expressed as cell count per microliter peripheral blood and are representative of three independent experiments (n = 6 mice/group/time point). Green diamonds- Sham Controls, Blue squares–BW, Red triangles–CP treatment (positive control). Asterisk indicates statistical significance (*p* < 0.05, ANOVA and Dunnett’s post-test with sham treated mice as control group).

Measuring the blood cell populations longitudinally following BW revealed a significant reduction in the absolute numbers of circulating immune cells up to 14 days post exposure (Figure 1). In addition, significant decreases were measured in the number of red blood cells and consequent hematocrit were observed throughout the follow up period. Even though the absolute number of different cell types changed, the composition of these cell types in the blood were not significantly altered by the BW exposure (data not presented).

### Changes in Viability of Cells in Primary and Secondary Immune Organs

**FIGURE 2:**
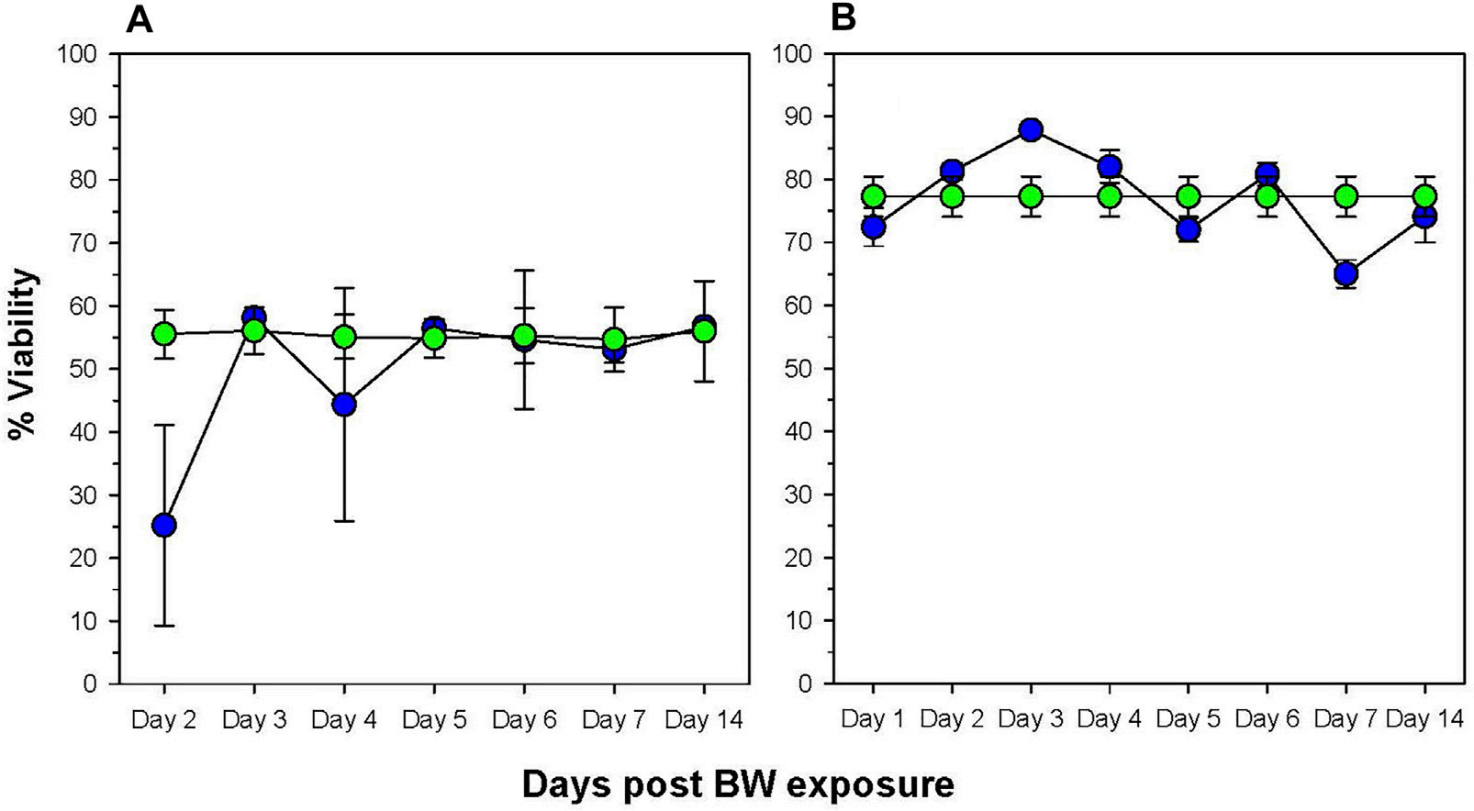
Changes in viability of cells in primary and secondary immune organs. The viability of bone marrow cells **(A)** and splenocytes **(B)** was assessed by staining cells with the viability dye 7-AAD and analyzing by flow cytometry. Data expressed as percentage viable cells within cell suspension (data representative of three separate experiments, n = 6 mice/group/time point). Blue = BW, green = sham control.

The next step was to identify potentially affected organs that may have contributed to the change in the absolute cell numbers in the blood. We speculate the potential explanations are phenotypic shift resulting from the pressure of the BW, apoptosis, or migration of cells from the blood into tissues. To assess cell viability, we examined the bone marrow, the primary immune organ, and the spleen, a secondary immune organ, where leukocytes will regularly pass through (Figure 2). Reflecting the decrease in the number of leukocytes in the blood on Day 1 and 2, we observed a significant decrease in viability in both splenocytes and bone marrow cells (*p* < 0.05). Viability in the bone marrow is significantly reduced on Day 2 (*p* = 0.002, Dunnett’s test), recovering to levels of sham animals on Day 3. The viability of splenocytes increased significantly on Day 2 and reaches a peak on Day 3 suggesting the induction of anti-apoptotic genes. There was another significant drop in viability on Day 7 (*p* = 0.01). Viability in the bone marrow was significantly reduced on Day 2 (*p* = 0.002, Dunnet’s test), recovering to levels of sham animals on Day 3.

### Blast Wave Exposure Results in Changes in the T-Helper Compartment of the Spleen

**FIGURE 3:**
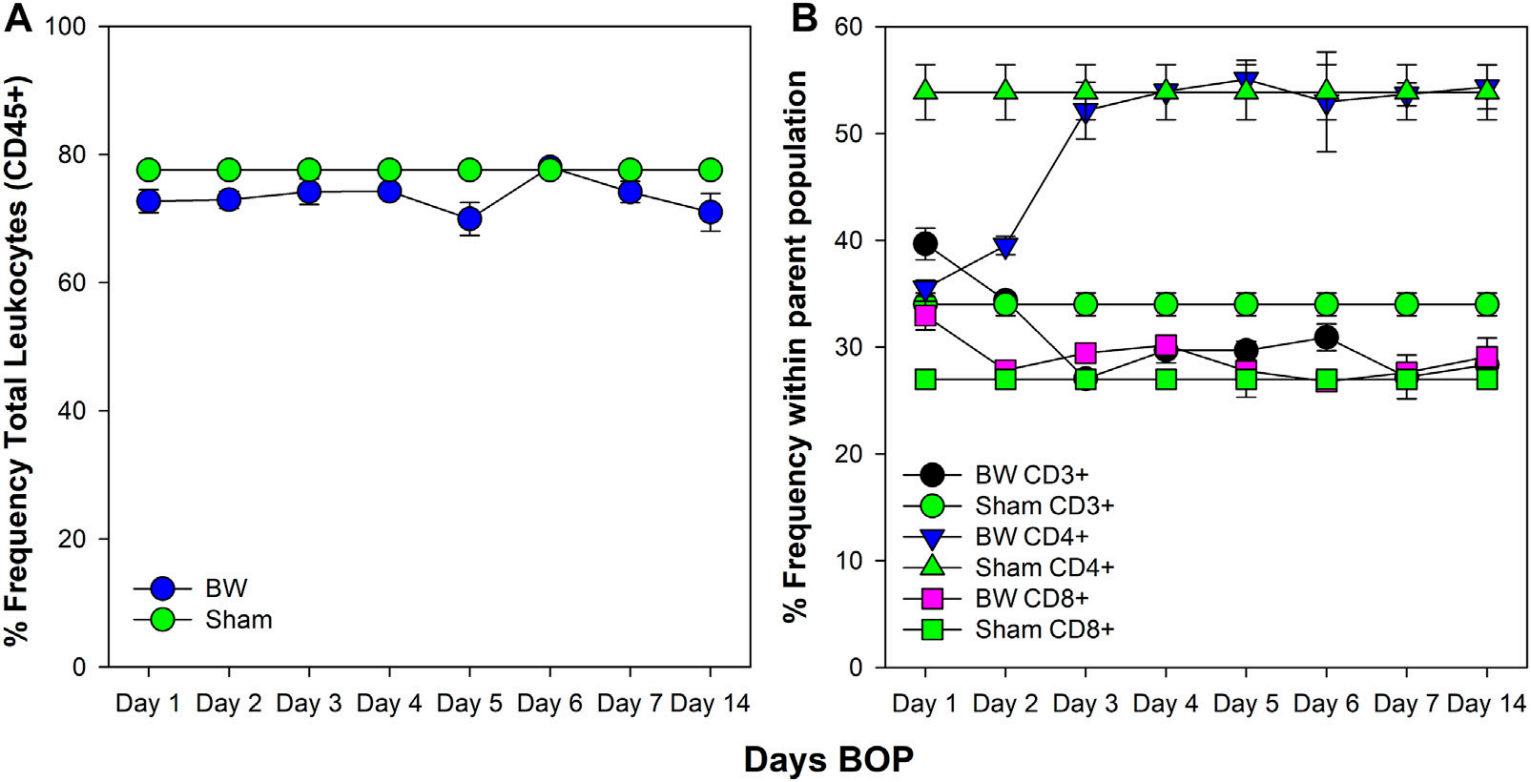
Changes in the frequency of leukocytes and T cell subsets in the spleen induced by BW. Frequency of viable leukocytes **(A)** and T cell subsets **(B)** was assessed by flow cytometry. Data from sham-treated animals were plotted as reference for each time point. Data representative of three experiments (n = 5 mice/group/time point).

The spleen is the main immune organ for the induction of humoral immune responses, especially those directed against bacteria (Leitner et al., 1994) and, therefore, changes induced by BW may have had significant impact on potential wound infections. The drop in the peripheral blood WBC count was paralleled by an initial drop in the total frequency of leukocytes in the spleen (Figure 3). This drop was significant for CD3^+^ and CD4^+^ T cells for the first 2 days after BW (*p* < 0.001, Dunnett’s test), recovering to the levels of sham mice thereafter. However, no changes were observed in CD8^+^ T cells (Figure 3B).

### Blast Wave Exposure Alters Viability of Hematopoietic Cells in the Bone Marrow

To assess the impact of BW on the bone marrow, based upon the changes observed in the peripheral blood, we used flow cytometric panels that differentiate between hematopoietic stem cells (HSC) [32, 33] and various multipotent progenitor cells (Table 1). Cohorts of animals were euthanized at the established longitudinal time points and bone marrow cells were isolated and analyzed.

**TABLE 1:** Flow cytometric panel for the identification of hematopoietic bone marrow populations.

Bone marrow population	Designation	Surface markers	Flow staining
Long-term Hematopoietic stem cells (HSC)	HSC	CD45^−^CD48^+^CD150^+^EPCR^-^	Viability
			CD45, CD48, CD150, EPCR
Hematopoietic stem cells expressing SLAM markers	ESLAM	CD45^+^CD48^+^CD150^+^EPCR^+^	Viability
			CD45, CD48, CD150, EPCR
Pluripotent and Hematopoetic stem cells	LSK/SLAM	Lin^+^Sca1^+^c-Kit^+^ CD48^+^CD150^+^	Viability
			Lineage markers (=CD3, CD11b, CD45R, Gr-1, Ter119)
			Sca1, c-Kit, CD48, CD150
Pluripotent hematopoietic cells	LSK	Lin^+^Sca1^+^c-Kit^+^	Viability
			Lineage markers (=CD3, CD11b, CD45R, Gr-1, Ter119)
			Sca1, c-Kit

**FIGURE 4:**
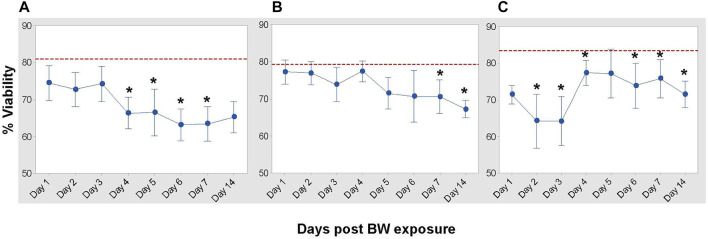
Impact of blast overpressure on viability of bone marrow populations. Viability of hematopoietic cells **(A)**, pluripotent progenitor cells (LSK/SLAM), **(B)**, and multi-potent bone marrow cells (LSK), **(C)** assessed longitudinally after BW using flow cytometry. Data expressed as mean viability ±SD of six mice/group/time point and representative of two separate experiments. Dotted line indicates viability of cell population in sham control mice. Asterisk indicates statistical significance (*p* < 0.05, ANOVA and Dunnett’s post-test with sham treated mice as control group).

Viability assessment demonstrated the impact of BW on total BMCs (Figure 4). Viability progressively decreased in HSC and pluripotent progenitor cells (Figure 4A, B). While there was an initial decrease in the viability of multi-potent progenitor cells (LSK) on Day 2 and 3 after BW, there was a significant increase in viability by Day 4 (Figure 4C).

### Blast Wave Exposure Results in Changes in the Frequency and Composition of Hematopoietic Cells in the Bone Marrow

**FIGURE 5:**
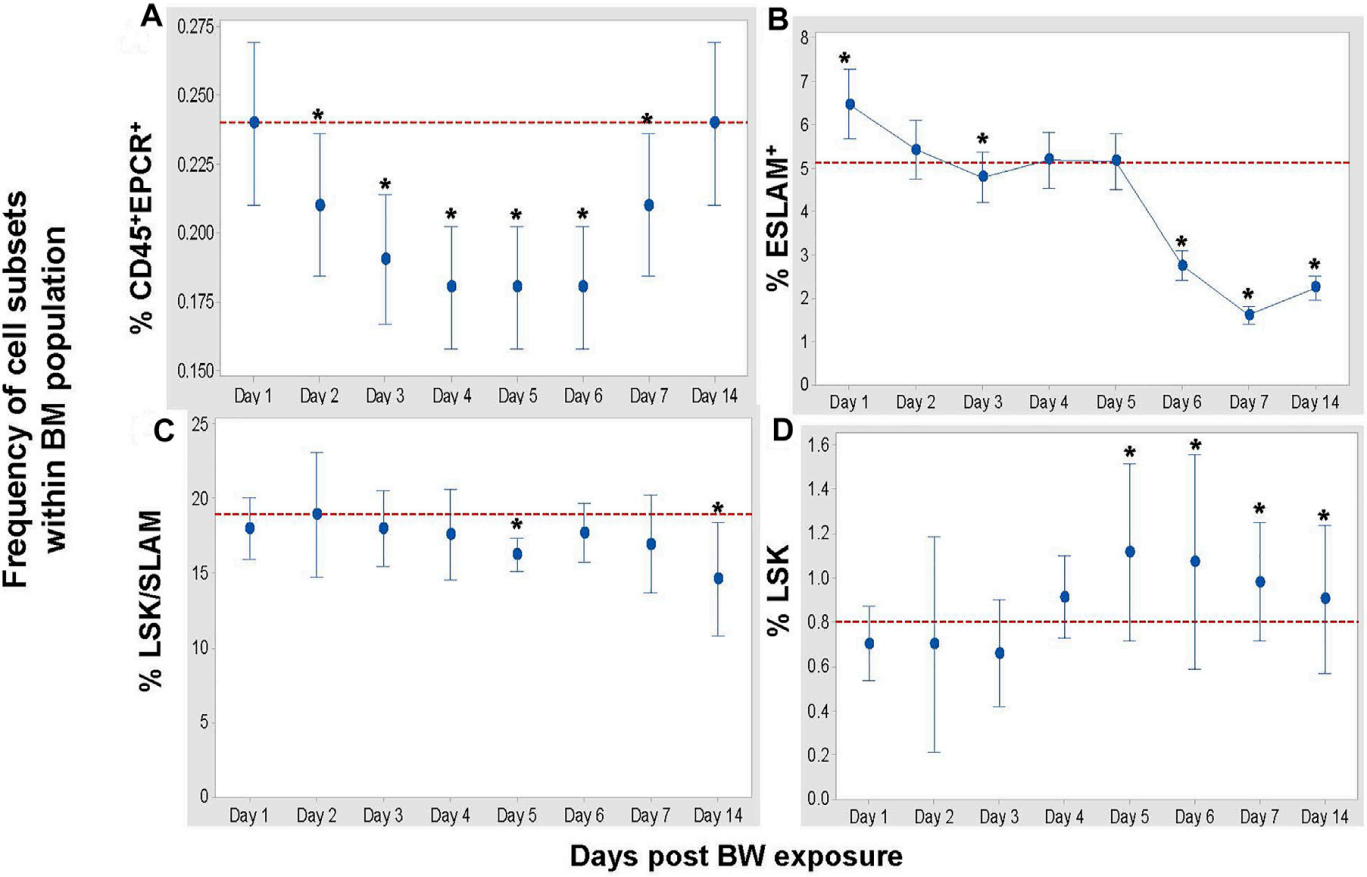
BW causes changes in the frequencies of hematopoietic stem cells (HSC) and progenitor cells. Data expressed as mean frequency of hematopoietic stem cells **(A)**, ESLAM + HSC **(B)**, LSK/SLAM cell subset **(C)**, and LSK cell subset **(D)** within parental bone marrow cells (Table 1). Representative experiment (n = 4 mice/group/time point, n = 8 sham control mice) of two separate experiments. Asterisk indicates statistical significance (*p* < 0.05, ANOVA and Dunnett’s post-test with sham treated mice as control group).

To assess whether there is a general or selective impact on HSC and pluri-/multi-potent progenitor cell subsets in the bone marrow, three distinct flow cytometric panels (Table 1) were applied for the in-depth analysis (Figure 5). BW differentially affects these cell populations in the following manner: First, the frequency of CD45^+^EPCR^+^ HSC decreased significantly on Days 3–6 (*p* < 0.001 Dunnett’s test) and slowly increased again to reach the levels of sham mice by Day 14 (Figure 5A). Second, the frequency of ESLAM^+^ HSC increased significantly after BW compared to the levels in bone marrow of sham mice, after which a significant drop in the frequency was observed starting on Day 6. A continued decline was observed lasting until the end of the experiment (Day 14). Third, changes in the LSK/SLAM subset were less pronounced except for significant decreases in the frequencies on day 5 and Day 14 (Figure 5C). Forth, the frequency of the LSK subset showed slight decreases in the initial days after BW preceding a marked increase on Day 4 that reached statistical significance on Days 5–7 (Figure 5D). We conclude that the impact of BW differed depending on the commitment level of the progenitor cells and subsets of HSC at the time of exposure, and also potentially involves altered phenotypical response from the exposure. It also becomes apparent that even after 14 days, there are still cellular changes occurring and the down-stream impact on the functionality and the homeostasis within the bone marrow remains unclear.

### Blast Wave Exposure Results in Sub-Acute Changes in the Cytokine/Chemokine Profile.

**FIGURE 6:**
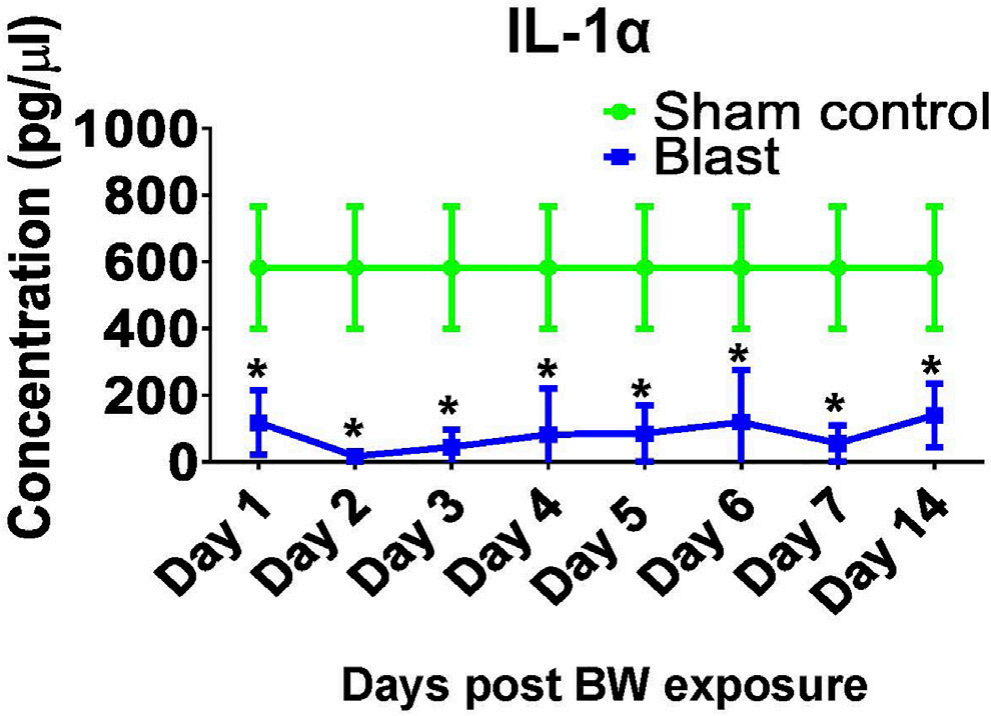
Changes in systemic IL-1α after BW. IL-1α was measured in sera from mice either sham or exposed to BW using a quantitative ELISA. Data expressed as mean pg/ml ± SD of n = 4 mice/group/time point. Asterisk indicates statistical significance (*p* < 0.05, ANOVA and Dunnett’s post-test with sham treated mice as control group).

Profiling of BW-induced changes was extended to assessments of the concentrations of cytokines and chemokines in serum of treated mice. IL-1α is one of the early pro-inflammatory cytokines and has an important role in the recruitment of immune cells to the injury site. Our results revealed that IL-1α was significantly reduced up to 14 days post blast, such that its concentration was 5–20 times lower in BW group than the sham controls (*p* < 0.05, *t*-test, Figure 6). IL-1α findings prompted an in-depth longitudinal analysis of cytokine profiles in mice exposed to BW compared to sham controls. Cytokine profiles in sera are summarized in Supplementary Table S1.

**FIGURE 7:**
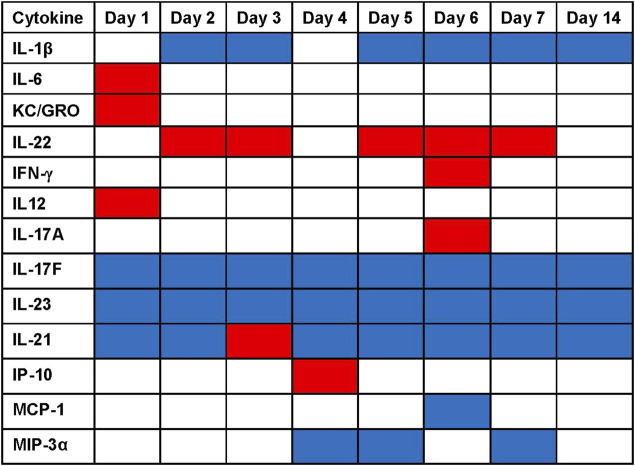
Significant changes in serum cytokine/chemokine levels after blast overpressure. Statistical differences (ANOVA, Dunnett’s test) between the serum concentrations of BW-treated vs. sham-treated mice are presented as heat map: significantly higher cytokine/chemokine levels in the BW group compared to control are indicated in red, significantly lower cytokine/chemokine levels are indicated in blue.

The statistical analysis comparing serum factor levels in BW-treated vs. sham-treated control mice (Figure 7) unveiled the following findings: 1) BW induced a largely pro-inflammatory milieu with significant increases of IL-6 IL-12, and KC/GRO on Day 1 and IL-22 on Days 2, 3, 5, 6, 7 (*p* < 0.001). Interestingly, IL-1β was significantly lower in the BW-treated animals on Days 2, 3, 5–14. Th1-cytokines remained un-affected by BW except for IL-12 on Day 1 and IFN-γ on Day 6 where a significant spike in serum cytokine levels was observed. 2) Th2- cytokines in serum were not impacted by BW. 3) Th-17 serum cytokines, namely IL-17F and IL-23 were significantly reduced after BW and there was a spike in IL-17A on Day 6. 4) The serum levels of regulatory IL-21 were significantly lower after BW except for the spike on Day 3. 5) The impact of serum levels of chemokines was surprisingly moderate: levels of IP-10 were significantly higher compared to controls on Day 4, and the levels of MCP-1 and MIP-3α were significantly lower on Day 6 and Days 4, 5, and 7, respectively.

## Discussion

The current study is, to the best of our knowledge, the first study to investigate long-term effects of blast overpressure on the immune system in peripheral blood, spleen, and at the bone marrow level. Our results suggest that BW exposure causes changes in the viability and frequency of bone marrow hematopoietic cells that translate into decreases in the number of peripheral white blood cells, red blood cells, and platelets. Such changes in the frequencies of innate and adaptive immune cells may be at least in part responsible for the reported susceptibility to wound infections and therefore impact health outcomes after blast-induced injuries. We observed changes in concentrations of cytokines and chemokines involved in the regulation of wound healing and inflammation as well as those that have important roles in infection/infection progression and hemorrhage control. Our results indicate that BW exposure leads to reduced functionality of the innate immune system and include significant reductions of (a) total WBC count including neutrophils, eosinophils and monocytes; (b) platelets which may have an impact if there are open injuries; (c) serum levels of chemokines (e.g. IL-1α,MIP-3α, MCP-1), which are necessary for the recruitment of immune cells fighting pathogens that enter the site through injury.

WBCs are critical to protect against infections (Shi and Pamer, 2011; Rosenberg et al., 2013; Rosales, 2018). Decreases in the frequencies of these cell populations as result of BW may be a significant pre-disposing factor for development of post-traumatic infections. Moreover, thrombocytopenia is another pre-disposing factor to infections as thrombocytes can fight infections alone or via interaction with other immune cells such as neutrophils and monocytes/macrophages (Martinod and Deppermann, 2021). Indeed, blast-related combat casualties showed very high occurrence of wide-spectrum wound infections (Thompson et al., 2019). Importantly, blast injury was highly coincidental with emergence of invasive fungal infections (IFIs) that caused high morbidity and mortality of combat casualties - 98% of all patients with IFIs were injured by blast (Warkentien et al., 2012; Tribble and Rodriguez, 2014; Ganesan et al., 2019). Our data also show a significant neutropenia, which is one of the major risk factors for development of IFI such as mucormycosis and other infections. IL-1α is one of the early pro-inflammatory cytokines that is involved in recruitment of immune cells to the site of infection and it is important in protection against various pathogens (Malik and Kanneganti, 2018). This cytokine has been shown to be critical for resistance to and clearance of highly virulent *P. aeruginosa* (Al Moussawi and Kazmierczak, 2014) and *A. fumigatus* strains (Caffrey-Carr et al., 2017) in animal models of lung infections. In addition to immune cell recruitment, cytokines also show direct antimicrobial effects; notably, MIP-3α (Yang et al., 2003) and other cytokines have been shown to kill various bacterial and fungal pathogens *in vitro*.

There is a clear indication of reduced functionality of the adaptive immune system due to a significant reduction of the frequency of CD4^+^ T cells in the spleen. The spleen is an important organ for the induction of humoral responses, especially against bacteria. CD4^+^ T cells are required in most cases to start an efficacious adaptive immune response by fully activating CD8^+^ T cells and B-cells (Han et al., 2008; Vitry et al., 2014; Loomis et al., 2020). Our data demonstrate a reduced viability of cell populations in the spleen and bone marrow after BW. There was a clear distinction between the various bone marrow cell populations at the level of the HSC and pluripotent progenitor cells with distinct patterns in the changes of the frequencies induced by BW. The question remains whether the reduction in cell numbers within the various BM populations is associated with apoptosis or with altered differentiation/commitment to specific lineages. If the reduction/depletion of stem cells and pluripotent progenitor cells is due to BW-induced differentiation of these cells into blood cells with limited life spans then this will ultimately lead to immunodeficiency, as peripheral immune cells cannot be replenished. This needs to be further investigated with transcriptomic studies and understand if this shift is transient or permanent. While the effects of a single BW exposure will lead to the reduction of hematopoietic stem and progenitor cells, it remains to be seen what the long-term effects are on the immune system and its fitness. Future work will conduct functional assays to assess the ability to fight wound infections and to determine how far these BW-induced deficiencies reach.

Early events after injury involve the recruitment of cells involved in wound healing through production of IL-1α,IL-1β, IL-6, and TNF-α (Wahl, 1994; Braddock, 2001; Gillitzer and Goebeler, 2001; Heldin and Birkhauser, 2001; Prince et al., 2020). Potential impediment of the immune system and wound healing may be forecasted by the cytokine profiles we observed, since serum levels of IL-1α and IL-1β were statistically significantly lower following blast exposure. The higher levels of IL-6 and KC/GRO may be triggered through the decrease of blood cells in the periphery and in the spleen in an effort to achieve homeostasis.

Restoration of damaged tissue and wound healing is mediated by the Th17 cytokine group, which recruit immune cells and granulocytes (McCartney-Francis et al., 2001). Th17 cytokines are significantly lower throughout the time points, potentially preventing proper healing since the inflammation may not be mitigated to physiological levels to establish a regenerating milieu. Lower levels of IL-21 may account for the persistence of the pro-inflammatory milieu.

One aspect of the study was to determine whether changes in systemic cytokines could be correlated with changes in the frequencies of different immune cell and bone marrow populations. The dependence of granulocytes such as neutrophils on IL-17A for functionality has been described (Liu et al., 2016). Our data raise the question whether the significant reduction in IL-17 contributes to the significantly lower frequencies of neutrophils and eosinophils in peripheral blood (Figure 1). Future work will determine whether cytokine treatment after BW could stabilize the frequency of granulocytes in the blood as a first approach to identify interventions.

A crucial role of IL-17 in hematopoiesis has been described (Krstic et al., 2012; Mojsilović et al., 2015) and a decrease in the systemic concentration of Th17 cytokines could explain the reduction in the frequencies of ESLAM, LSK/SLAM, and LSK subpopulations. Th2 cytokines such as IL-4 affect the maturation of certain immune cell populations (Wang et al., 2015); since no changes in the systemic levels of these Th2 cytokines was induced by BW, we do not anticipate that the maturation of cell populations such as dendritic cells would be impaired. These differential changes in systemic cytokine levels suggest a target cell specific response of immune cells to BW. Future studies will dissect the impact of BW on the functionality of various immune cells and the resulting ability to fight infections.

The unique changes in the profile serum cytokine levels caused by BW are not unexpected considering the diverse functions of the respective cytokines and their role in immune defense against various pathogens. To our knowledge this study establishes for the first time the impact of a non-infectious stimulus, i.e., BW, on serum cytokine profiles. Similar to immunoprofiling responses to pathogens, the cellular source of the cytokines/chemokines remains elusive. While it would be desirable to identify the sources of these factors, identifying the triggers for these changes may be more attainable. Such triggers could be extensive cell death releasing DAMPs (Li et al., 2020), stress factors and/or hormones (Baxter et al., 2013), or triggering pressure sensing receptors (e.g. piezo-1 and piezo-2 receptors) (Keating and Cullen, 2021) in an attempt to unveil treatment targets.

The BW propagates throughout the entire body, inducing a wide myriad of changes in different organ systems and tissues (Cernak and Kobeissy, 2015). Taking this into account, there are several pathways that could potentially have roles in the observed effects of BW on the immune system and cause direct damage to the bone marrow cells and lymphoid organs. However, BW can also result in generation of free oxygen and nitrogen radicals throughout the body, including in tissues of the immune system, that can lead to damage to the immune cells and consequent changes in CBC and concentrations of cytokines and chemokines. Furthermore, a connection between the immune and nervous systems is well established (Maryanovich et al., 2018) and can be another means by which BW affects the immune system. Exposure to BW leads to varied degrees of traumatic brain injury and more specifically to changes in *vagus* nerve output. N. *vagus* has an essential role in innervation of the spleen (Bassi et al., 2020), immune cells in the gut (Matteoli and Boeckxstaens, 2013; Bonaz et al., 2017) and bone marrow (Maryanovich et al., 2018) and changes in *vagus* nerve output can be readily envisioned to contribute to the observed effects on the periphery such as recruitment and migration of immune cells. Moreover, we recently demonstrated that BW results in changes in the pharmacokinetics of cefazolin, first line of defense antibiotic in military patients, resulting in retention of antibiotic in liver and plasma and decreased concentration in target skin tissue (Antonic et al., 2020; Selig et al., 2021). Combined, these effects can lead to increased infection susceptibility and other complications.

In conclusion, we provided, to the best of our knowledge, results of the first study of long term effects of BW on the immune system and a potential mechanism of correlating of blast exposure to increased infection susceptibility. Our data suggest that BW exposure can directly and indirectly contribute to infection development through prolonged immune changes alongside altered pharmacokinetics of applied antibiotics by disturbing and dysregulating compartments of the immune system. Further analysis is needed to derive strong conclusions of immune compromise with techniques such as transcriptomic and mechanisms of cell death.

## Data Availability

The original contributions presented in the study are included in the article/Supplementary Material, further inquiries can be directed to the corresponding authors.

## Author Disclaimer

Material has been reviewed by the Walter Reed Army Institute of Research. There is no objection to its presentation and/or publication. This paper has been approved for public release with unlimited distribution. The investigators have adhered to the policies for protection of human subjects as prescribed in AR 70–25. BS, ST, DG are a military service members, EB-L, JL, VSS are government employees and this work was prepared as part of her official duties. Title 17 U.S.C. § 105 provides that copyright protection under this title is not available for any work of the U.S. Government. Title 17 U.S.C. § 105 defines a U.S. Government work as a work prepared by a military service member or employee of the U.S. Government as part of that person’s official duties. The views expressed in this article are those of the authors and do not necessarily reflect the official policy or position of the Department of the Navy, Department of the Army, Department of Defense, the U.S. Government, nor The HM. Jackson Foundation for the Advancement of Military Medicine, Inc. (HJF).

## References

[B1] Al Moussawi K. Kazmierczak B. I. (2014). Distinct Contributions of Interleukin-1α (IL-1α) and IL-1β to Innate Immune Recognition of *Pseudomonas aeruginosa* in the Lung. Infect. Immun. 82 (10), 4204–4211. 10.1128/iai.02218-14 25069982 PMC4187872

[B2] Antonic V. Sajja V. Sousa J. Nguyen K. Alamneh Y. Garry B. (2020). Evaluation of Blast Overpressure Exposure Effects on Concentration of Antibiotics in Mice. Mil. Med. 185 (Suppl. 1), 256–262. 10.1093/milmed/usz212 32074328

[B3] Army U. S. (2018). The U.S. Army in Multi-Domain Operations 2028. TRADOC Pamphlet 525, 3-1.

[B4] Bala M. Rivkind A. I. Zamir G. Hadar T. Gertsenshtein I. Mintz Y. (2008). Abdominal Trauma after Terrorist Bombing Attacks Exhibits a Unique Pattern of Injury. Ann. Surg. 248 (2), 303–309. 10.1097/SLA.0b013e318180a3f7 18650642

[B5] Bassi G. S. Kanashiro A. Coimbra N. C. Terrando N. Maixner W. Ulloa L. (2020). Anatomical and Clinical Implications of Vagal Modulation of the Spleen. Neurosci. Biobehavioral Rev. 112, 363–373. 10.1016/j.neubiorev.2020.02.011 PMC7211143 32061636

[B6] Baxter D. Sharp D. J. Feeney C. Papadopoulou D. Ham T. E. Jilka S. (2013). Pituitary Dysfunction after Blast Traumatic Brain Injury. Ann. Neurol. 74 (4), 527–536. 10.1002/ana.23958 23794460 PMC4223931

[B7] Blyth D. M. Yun H. C. Tribble D. R. Murray C. K. (2015). Lessons of War. J. Trauma Acute Care Surg. 79 (4), S227–S235. 10.1097/ta.0000000000000768 26406435 PMC4586048

[B8] Bonaz B. Sinniger V. Pellissier S. (2017). The Vagus Nerve in the Neuro-Immune Axis: Implications in the Pathology of the Gastrointestinal Tract. Front. Immunol. 8, 1452. 10.3389/fimmu.2017.01452 29163522 PMC5673632

[B9] Braddock M. (2001). The Transcription Factor Egr-1: a Potential Drug in Wound Healing and Tissue Repair. Ann. Med. 33, 313–318. 10.3109/07853890109002083 11491188

[B10] Burns T. C. Stinner D. J. Mack A. W. Potter B. K. Beer R. Eckel T. T. (2012). Microbiology and Injury Characteristics in Severe Open Tibia Fractures from Combat. J. Trauma Acute Care Surg. 72 (4), 1062–1067. 10.1097/TA.0b013e318241f534 22491628

[B11] Caffrey-Carr A. K. Kowalski C. H. Beattie S. R. Blaseg N. A. Upshaw C. R. Thammahong A. (2017). Interleukin 1α Is Critical for Resistance against Highly Virulent Aspergillus fumigatus Isolates. Infect. Immun. 85 (12). 10.1128/iai.00661-17 PMC5695118 28947643

[B12] Cernak I. Savic J. Malicevic Z. Zunic G. Radosevic P. Ivanovic I. (1996). Involvement of the central Nervous System in the General Response to Pulmonary Blast Injury. J. Trauma 40 (3 Suppl. l), S100–S104. 10.1097/00005373-199603001-00023 8606388

[B13] Cernak I. (2015). “Frontiers in Neuroengineering. Blast Injuries and Blast-Induced Neurotrauma: Overview of Pathophysiology and Experimental Knowledge Models and Findings,” in Brain Neurotrauma: Molecular, Neuropsychological, and Rehabilitation Aspects. Editor Kobeissy F. H. (Boca Raton (FL: CRC Press/Taylor & Francis, © 2015 by Taylor & Francis Group, LLC.). 26269895

[B14] Cernak I. (2010). The Importance of Systemic Response in the Pathobiology of Blast-Induced Neurotrauma. Front. Neur. 1, 151. 10.3389/fneur.2010.00151 PMC3009449 21206523

[B15] Cho H. J. Sajja V. S. S. S. Vandevord P. J. Lee Y. W. (2013). Blast Induces Oxidative Stress, Inflammation, Neuronal Loss and Subsequent Short-Term Memory Impairment in Rats. Neuroscience 253, 9–20. 10.1016/j.neuroscience.2013.08.037 23999126

[B16] Cripps N. P. J. Cooper G. J. (1997). Risk of Late Perforation in Intestinal Contusions Caused by Explosive Blast. Br. J. Surg. 84 (9), 1298–1303. 10.1046/j.1365-2168.1997.02790.x 9313719

[B17] D'Alleyrand J.-C. G. Lewandowski L. R. Forsberg J. A. Gordon W. T. Fleming M. E. Mullis B. H. (2015). Combat-Related Hemipelvectomy. J. Orthop. Trauma 29 (12), e493–e498. 10.1097/bot.0000000000000398 26595599

[B18] Dougherty A. L. Mohrle C. R. Galarneau M. R. Woodruff S. I. Dye J. L. Quinn K. H. (2009). Battlefield Extremity Injuries in Operation Iraqi Freedom. Injury 40 (7), 772–777. 10.1016/j.injury.2009.02.014 19450798

[B19] Elsayed N. M. Gorbunov N. V. (2007). Pulmonary Biochemical and Histological Alterations after Repeated Low-Level Blast Overpressure Exposures. Toxicol. Sci. 95 (1), 289–296. 10.1093/toxsci/kfl138 17060374

[B20] Ganesan A. Shaikh F. Bradley W. Blyth D. M. Bennett D. Petfield J. L. (2019). Classification of Trauma-Associated Invasive Fungal Infections to Support Wound Treatment Decisions. Emerg. Infect. Dis. 25 (9), 1639–1647. 10.3201/eid2509.190168 PMC6711217 31441428

[B21] Gillitzer R. Goebeler M. (2001). Chemokines in Cutaneous Wound Healing. J. Leukoc. Biol. 69 (4), 513–521. 10.1189/jlb.69.4.513 11310836

[B22] Han S. Norimine J. Palmer G. H. Mwangi W. Lahmers K. K. Brown W. C. (2008). Rapid Deletion of Antigen-specific CD4+T Cells Following Infection Represents a Strategy of Immune Evasion and Persistence forAnaplasma Marginale. J. Immunol. 181 (11), 7759–7769. 10.4049/jimmunol.181.11.7759 19017965 PMC2815346

[B23] Health C. o. G. W. a. (2014). “Long-Term Effects of Blast Exposures; Board on the Health of Select Populations; Institute of Medicine. Gulf War and Health, Volume 9: Long-Term Effects of Blast Exposures,” in Pathophysiology of Blast Injury and Overview of Experimental Data (Washington (DC): National Academies Press). 24872971

[B24] Heldin C. H. (2001). “Signal Transduction Mechanisms for Members of the TGF-Beta Family,” in TGF-beta and Related Cytokines in Inflammation. Editor Birkhauser S. N. a. S. M. W. Basel.

[B25] Hospenthal D. R. Murray C. K. Andersen R. C. Bell R. B. Calhoun J. H. Cancio L. C. (2011). Guidelines for the Prevention of Infections Associated with Combat-Related Injuries: 2011 Update. J. Trauma 71 (2 Suppl. 2), S210–S234. 10.1097/TA.0b013e318227ac4b 21814089

[B26] Hospenthal D. R. Murray C. K. (2011). Preface: Guidelines for the Prevention of Infections Associated with Combat-Related Injuries: 2011 Update. J. Trauma 71 (2 Suppl. 2), S197–S201. 10.1097/TA.0b013e318227ac23 21814087

[B27] Huyan X.-H. Lin Y.-P. Gao T. Chen R.-Y. Fan Y.-M. (2011). Immunosuppressive Effect of Cyclophosphamide on white Blood Cells and Lymphocyte Subpopulations from Peripheral Blood of Balb/c Mice. Int. Immunopharmacology 11 (9), 1293–1297. 10.1016/j.intimp.2011.04.011 21530682

[B28] Jacobs A. C. Thompson M. G. Black C. C. Kessler J. L. Clark L. P. McQueary C. N. (2014). AB5075, a Highly Virulent Isolate of Acinetobacter Baumannii, as a Model Strain for the Evaluation of Pathogenesis and Antimicrobial Treatments. mBio 5 (3), e01076–14. 10.1128/mBio.01076-14 24865555 PMC4045072

[B29] Kaba S. A. Karch C. P. Seth L. Ferlez K. M. B. Storme C. K. Pesavento D. M. (2018). Self-assembling Protein Nanoparticles with Built-In Flagellin Domains Increases Protective Efficacy of a Plasmodium Falciparum Based Vaccine. Vaccine 36 (6), 906–914. 10.1016/j.vaccine.2017.12.001 29269157

[B30] Keating C. E. Cullen D. K. (2021). Mechanosensation in Traumatic Brain Injury. Neurobiol. Dis. 148, 105210. 10.1016/j.nbd.2020.105210 33259894 PMC7847277

[B31] Kirkman E. Watts S. (2011). Characterization of the Response to Primary Blast Injury. Phil. Trans. R. Soc. B 366 (1562), 286–290. 10.1098/rstb.2010.0249 21149364 PMC3013437

[B32] Koliatsos V. E. Cernak I. Xu L. Song Y. Savonenko A. Crain B. J. (2011). A Mouse Model of Blast Injury to Brain: Initial Pathological, Neuropathological, and Behavioral Characterization. J. Neuropathol. Exp. Neurol. 70 (5), 399–416. 10.1097/NEN.0b013e3182189f06 21487304

[B33] Krstic A. Mojsilovic S. Jovcic G. Bugarski D. (2012). The Potential of Interleukin-17 to Mediate Hematopoietic Response. Immunol. Res. 52 (1), 34–41. 10.1007/s12026-012-8276-8 22392050

[B34] Kuriakose M. Rama Rao K. V. Younger D. Chandra N. (2018). Temporal and Spatial Effects of Blast Overpressure on Blood-Brain Barrier Permeability in Traumatic Brain Injury. Sci. Rep. 8 (1), 8681. 10.1038/s41598-018-26813-7 29875451 PMC5989233

[B35] Leitner W. Bergmann E. S. Thalhamer J. (1994). Regeneration of Splenic Stromal Elements. Res. Exp. Med. 194 (4), 221–230. 10.1007/bf02576383 7800931

[B36] Li N. Geng C. Hou S. Fan H. Gong Y. (2020). Damage-Associated Molecular Patterns and Their Signaling Pathways in Primary Blast Lung Injury: New Research Progress and Future Directions. Ijms 21 (17), 6303. 10.3390/ijms21176303 PMC7504526 32878118

[B37] Liu R. Lauridsen H. M. Amezquita R. A. Pierce R. W. Jane-Wit D. Fang C. (2016). IL-17 Promotes Neutrophil-Mediated Immunity by Activating Microvascular Pericytes and Not Endothelium. J.I. 197 (6), 2400–2408. 10.4049/jimmunol.1600138 PMC5010945 27534549

[B38] Liu X. Quan N. (2015). Immune Cell Isolation from Mouse Femur Bone Marrow. Bio-protocol 5 (20). 10.21769/bioprotoc.1631 PMC4948643 27441207

[B39] Loomis W. P. Delaney M. A. Johnson M. L. Cookson B. T. (2020). Failure of CD4 T Cell-Deficient Hosts to Control Chronic Nontyphoidal Salmonella Infection Leads to Exacerbated Inflammation, Chronic Anemia, and Altered Myelopoiesis. Infect. Immun. 89 (1). 10.1128/iai.00417-20 PMC7927941 33046510

[B40] Malik A. Kanneganti T.-D. (2018). Function and Regulation of IL-1α in Inflammatory Diseases and Cancer. Immunol. Rev. 281 (1), 124–137. 10.1111/imr.12615 29247991 PMC5739076

[B41] Martinod K. Deppermann C. (2021). Immunothrombosis and Thromboinflammation in Host Defense and Disease. Platelets 32 (3), 314–324. 10.1080/09537104.2020.1817360 32896192

[B42] Maryanovich M. Takeishi S. Frenette P. S. (2018). Neural Regulation of Bone and Bone Marrow. Cold Spring Harb Perspect. Med. 8 (9), a031344. 10.1101/cshperspect.a031344 29500307 PMC6119651

[B43] Matteoli G. Boeckxstaens G. E. (2013). The Vagal Innervation of the Gut and Immune Homeostasis. Gut 62 (8), 1214–1222. 10.1136/gutjnl-2012-302550 23023166 PMC3711371

[B44] McCartney-Francis N. L. Wahl S. M. (2001). “TGF-beta and Macrophages in the Rise and Fall of Inflammation,” in TGF-beta and Related Cytokines in Inflammation. Editor Birkhauser S. N. a. S. M. W. B. (Basel).

[B45] Melvin S. J. Dombroski D. G. Torbert J. T. Kovach S. J. Esterhai J. L. Mehta S. (2010). Open Tibial Shaft Fractures: I. Evaluation and Initial Wound Management. Am. Acad. Orthopaedic Surgeon 18 (1), 10–19. 10.5435/00124635-201001000-00003 20044487

[B46] Mojsilović S. Jauković A. Santibañez J. F. Bugarski D. (2015). Interleukin-17 and its Implication in the Regulation of Differentiation and Function of Hematopoietic and Mesenchymal Stem Cells. Mediators Inflamm. 2015, 1–11. 10.1155/2015/470458 PMC4427009 25999667

[B47] Murray C. K. (2008). Infectious Disease Complications of Combat-Related Injuries. Crit. Care Med. 36 (7 Suppl. l), S358–S364. 10.1097/CCM.0b013e31817e2ffc 18594263

[B48] office B. I. R. P. c. (2016). “Minimizing the Impact of Wound Infections Following Blast-Related Injuries,” in International Satte of the Science Meeting (Ft.Detrick: Dept of Defense).

[B49] Prince N. Penatzer J. A. Dietz M. J. Boyd J. W. (2020). Impact of Cytokines and Phosphoproteins in Response to Chronic Joint Infection. Biology 9 (7), 167. 10.3390/biology9070167 PMC7407198 32708756

[B50] Rosales C. (2018). Neutrophil: A Cell with Many Roles in Inflammation or Several Cell Types? Front. Physiol. 9, 113. 10.3389/fphys.2018.00113 29515456 PMC5826082

[B51] Rosenberg H. F. Dyer K. D. Foster P. S. (2013). Eosinophils: Changing Perspectives in Health and Disease. Nat. Rev. Immunol. 13 (1), 9–22. 10.1038/nri3341 23154224 PMC4357492

[B52] Rozen P. a. D. I. (2011). “Wound Ballistic and Tissue Damage,” in Armed Conflict Injuries to the Extremities. Editors Lerner A. a. S. (Dordrecht: Springer Heidelberg).

[B53] Sajja V. S. Statz J. K. Walker L. P. B. Gist I. D. Wilder D. M. Ahlers S. T. (2020). Pulmonary Injury Risk Curves and Behavioral Changes from Blast Overpressure Exposures of Varying Frequency and Intensity in Rats. Sci. Rep. 10 (1), 16644. 10.1038/s41598-020-73643-7 33024181 PMC7538583

[B54] Selig D. J. Chin G. C. Bobrov A. G. DeLuca J. P. Getnet D. Livezey J. R. (2021). Semimechanistic Modeling of the Effects of Blast Overpressure Exposure on Cefazolin Pharmacokinetics in Mice. J. Pharmacol. Exp. Ther. 379, 175–181. 10.1124/jpet.121.000797 34433578 PMC8626777

[B55] Sheppard F. R. Keiser P. Craft D. W. Gage F. Robson M. Brown T. S. (2010). The Majority of US Combat Casualty Soft-Tissue Wounds Are Not Infected or Colonized upon Arrival or during Treatment at a continental US Military Medical Facility. Am. J. Surg. 200 (4), 489–495. 10.1016/j.amjsurg.2010.03.001 20887842

[B56] Shi C. Pamer E. G. (2011). Monocyte Recruitment during Infection and Inflammation. Nat. Rev. Immunol. 11 (11), 762–774. 10.1038/nri3070 21984070 PMC3947780

[B57] Stewart L. Shaikh F. Bradley W. Lu D. Blyth D. M. Petfield J. L. (2019). Combat-Related Extremity Wounds: Injury Factors Predicting Early Onset Infections. Mil. Med. 184 (Suppl. 1), 83–91. 10.1093/milmed/usy336 30901441 PMC6432943

[B58] Surbatovic M. Filipovic N. Radakovic S. Stankovic N. Slavkovic Z. (2007). Immune Cytokine Response in Combat Casualties: Blast or Explosive Trauma with or without Secondary Sepsis. Mil. Med. 172 (2), 190–195. 10.7205/milmed.172.2.190 17357775

[B59] Technology S. (2018). Flow Cytometry Methods for Identifying Mouse Hematopoietic Stem and Progenitor Cells. Tech. Bull. DOCUMENT # 27103 VERSION 1.1.0 AUG 2018.

[B60] Thompson K. B. Krispinsky L. T. Stark R. J. (2019). Late Immune Consequences of Combat Trauma: a Review of Trauma-Related Immune Dysfunction and Potential Therapies. Mil. Med Res 6 (1), 11. 10.1186/s40779-019-0202-0 31014397 PMC6480837

[B61] Thompson M. G. Black C. C. Pavlicek R. L. Honnold C. L. Wise M. C. Alamneh Y. A. (2014). Validation of a Novel Murine Wound Model of Acinetobacter Baumannii Infection. Antimicrob. Agents Chemother. 58 (3), 1332–1342. 10.1128/AAC.01944-13 24342634 PMC3957858

[B62] Thompson M. G. Truong-Le V. Alamneh Y. A. Black C. C. Anderl J. Honnold C. L. (2015). Evaluation of Gallium Citrate Formulations against a Multidrug-Resistant Strain of *Klebsiella pneumoniae* in a Murine Wound Model of Infection. Antimicrob. Agents Chemother. 59 (10), 6484–6493. 10.1128/AAC.00882-15 26239978 PMC4576086

[B63] Tribble D. R. Rodriguez C. J. (2014). Combat-Related Invasive Fungal Wound Infections. Curr. Fungal Infect. Rep. 8 (4), 277–286. 10.1007/s12281-014-0205-y 25530825 PMC4267292

[B64] Tümer N. Svetlov S. Whidden M. Kirichenko N. Prima V. Erdos B. (2013). Overpressure Blast-Wave Induced Brain Injury Elevates Oxidative Stress in the Hypothalamus and Catecholamine Biosynthesis in the Rat Adrenal Medulla. Neurosci. Lett. 544, 62–67. 10.1016/j.neulet.2013.03.042 23570732

[B65] van H. Mason W. Damon E. G. Dickinson A. R. Nevison T. O. Jr. (1971). Arterial Gas Emboli after Blast Injury. Exp. Biol. Med. 136 (4), 1253–1255. 10.3181/00379727-136-35469 5554473

[B66] Vitry M.-A. Hanot Mambres D. De Trez C. Akira S. Ryffel B. Letesson J.-J. (2014). Humoral Immunity and CD4+Th1 Cells Are Both Necessary for a Fully Protective Immune Response upon Secondary Infection withBrucella Melitensis. J.I. 192 (8), 3740–3752. 10.4049/jimmunol.1302561 24646742

[B67] Wahl S. M. (1994). Transforming Growth Factor Beta: the Good, the Bad, and the Ugly. J. Exp. Med. 180 (5), 1587–1590. 10.1084/jem.180.5.1587 7964446 PMC2191721

[B68] Wang S. Sun X. Zhou H. Zhu Z. Zhao W. Zhu C. (2015). Interleukin-4 Affects the Mature Phenotype and Function of Rat Bone Marrow-Derived Dendritic Cells. Mol. Med. Rep. 12 (1), 233–237. 10.3892/mmr.2015.3349 25683957

[B69] Warkentien T. Rodriguez C. Lloyd B. Wells J. Weintrob A. Dunne J. R. (2012). Invasive Mold Infections Following Combat-Related Injuries. Clin. Infect. Dis. 55 (11), 1441–1449. 10.1093/cid/cis749 23042971 PMC3657499

[B70] Yang D. Chen Q. Hoover D. M. Staley P. Tucker K. D. Lubkowski J. (2003). Many Chemokines Including CCL20/MIP-3α Display Antimicrobial Activity. J. Leukoc. Biol. 74 (3), 448–455. 10.1189/jlb.0103024 12949249

