# Supplementary material for: Blast Waves Cause Immune System Dysfunction and Transient Bone Marrow Failure in a Mouse Model
Source: Front Bioeng Biotechnol. 2022 Mar 22;10:821169. doi: 10.3389/fbioe.2022.821169 (PMC8980552; doi:10.3389/fbioe.2022.821169)
Supplement: Supplementary file 1 [file DataSheet1.DOCX]

## Supplementary Figure 1 – Gating strategies for bone marrow cell population

**
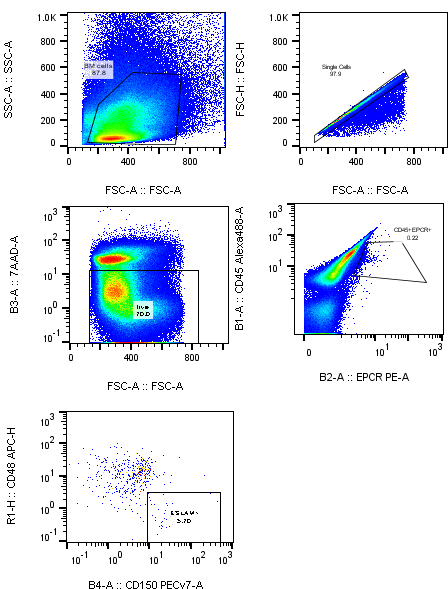
A**

**Supplementary Figure 1A .** Gating strategy of ESLAM cell subset. Cells were gated for bone marrow cells by Forward and Side scatter (FSC:SSC), then by single cells (FSC-A:FSC-H), then by viability (7AAD negative), and then based on lineage markers EPCR and CD45. ESLAM cells were quantified based on CD150^+^CD48^-^ phenotype.


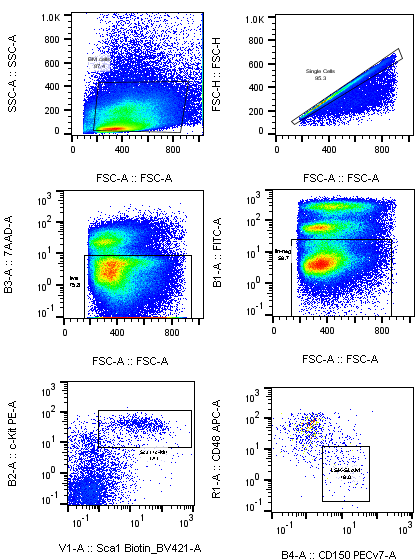


**Supplementary Figure 1B .** Gating strategy of LSK/SLAM cell subset. Cells were gated for bone marrow cells by Forward and Side scatter (FSC:SSC), then by single cells (FSC-A:FSC-H), then by viability (7AAD negative), and then based on lack of expression of lineage markers (lin^-^), then by expression of c-kit and Sca-1. LSK/SLAM cells were quantified based on a CD150^+^ CD48^-^ phenotype.


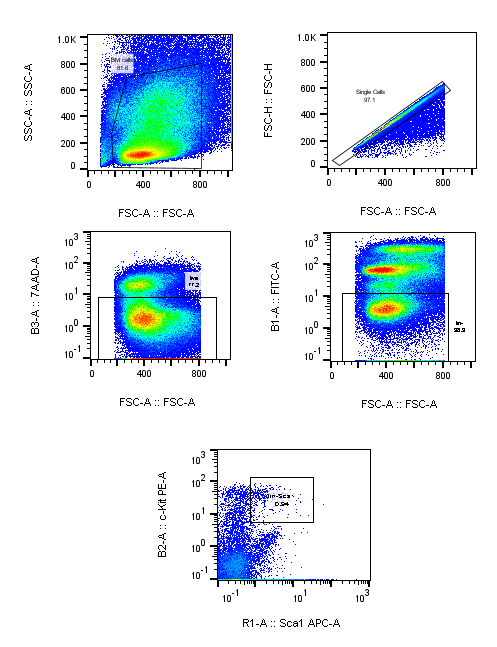


**Supplementary Figure 1C .** Gating strategy of LSK cell subset. Cells were gated for bone marrow cells by Forward and Side scatter (FSC:SSC), then by single cells (FSC-A:FSC-H), then by viability (7AAD negative), and then based on lack of expression of lineage markers (lin^-^). LSK cells were quantified based on expression of c-kit and Sca-1 (c-kit^+^ Sca1^+^).

***Supplementary Table 1: Serum levels of cytokines/chemokines significantly impacted by blast overpressure ^a^***

| Factor | Sham | Day 1 | Day 2 | Day 3 | Day 4 | Day 5 | Day 6 | Day 7 | Day 14 |
| --- | --- | --- | --- | --- | --- | --- | --- | --- | --- |
| IL-1β | 4.4 ± 1.4 | 2.4 ± 0.6 | 0.87 ± 0.2 | 0.81 ± 0.3 | 2.38 ± 0.4 | 0.94 ± 0.4 | 0.72 ± 0.1 | 1.1 ± 0.2 | 1.5 ± 0.4 |
| IL-6 | 7.4 ± 0.5 | 25.9 ± 4.8 | 16.1 ± 5.2 | 10.4 ± 4.3 | 9.4 ± 2.0 | 9.3 ± 2.3 | 12.9 ± 3.9 | 4.7 ± 1.6 | 4.5 ± 1.1 |
| KC/GRO | 62.5 ± 13.6 | 98.9 ± 20.2 | 56.6 ± 8.6 | 43.6 ± 7.7 | 70.3 ± 21.9 | 49.0 ± 5.8 | 38.1 ± 3.3 | 69.6 ± 9.3 | 57.0 ± 4.1 |
| IL-22 | 4.9 ± 0.8 | 3.70 ± 0.8 | 27.8 ± 18.4 | 13.0 ± 2.2 | 5.3 ± 1.9 | 12.6 ± 2.3 | 16.3 ± 3.0 | 15.3 ± 2.4 | 6.4 ± 1.7 |
| IFN-γ | 0.43 ± 0.02 | 0.66 ± 0.06 | 0.69 ± 0.2 | 0.82 ± 0.2 | 0.54 ± 0.05 | 0.63 ± 0.06 | 1.1 ± 0.17 | 0.72 ± 0.07 | 0.58 ± 0.04 |
| IL-12 | BD^b^ | 12.1 ± 5.6 | BD | 0.85 ± 0.1 | 3.46 ± 0.4 | 4.5 ± 0.3 | BD | BD | BD |
| IL-17A | 0.21 ± 0.09 | 0.15 ± 0.1 | 0.16 ± 0.08 | 0.19 ± 0.1 | BD | 0.23 ± 0.1 | 1.3 ± 0.4 | 0.02 ± 0.02 | 0.08 ± 0.04 |
| IL-17F | 2.9 ± 0.2 | BD | BD | BD | BD | BD | BD | BD | BD |
| IL-23 | 3.4 ± 0.5 | BD | BD | 0.29 ± 0.03 | BD | 0.83 ± 0.05 | BD | BD | 1.0 ± 0.04 |
| IL-21 | 0.7 ± 0.3 | BD | BD | 1.5 ± 0.9 | BD | 0.03 ± 0.03 | BD | BD | 0.14 ± 0.14 |
| IP-10 | 43.8 ± 0.4 | 48.2 ± 2.5 | 45.7 ± 1.8 | 49.8 ± 5.2 | 32.3 ± 3.1 | 45.5 ± 4.6 | 40.4 ± 3.6 | 36.5 ± 4.8 | 38.4 ± 1.1 |
| MCP-1 | 15.1 ± 0.6 | 17.8 ± 4.0 | 14.3 ± 0.9 | 11.7 ± 1.6 | 14.4 ± 2.4 | 11.3 ± 0.9 | 8.6 ± 1.4 | 16.2 ± 2.4 | 11.7 ± 0.4 |
| MIP-3α | 56.0 ± 7.9 | 49.0 ± 5.4 | 41.5 ± 2.7 | 49.5 ± 9.7 | 33.4 ± 2.5 | 38.0 ± 2.7 | 43.0 ± 5.0 | 43.7 ± 7.1 | 48.2 ± 3.3 |

^a^ Data expressed as mean pg/ml (± SD) of n=4 mice/time point

^b^ Below detection (BD). Detection limit according to manufacturer’s data sheet: IL12p70: 48pg/ml, IL17F 24 pg/ml, IL-23 4.9 pg/ml, IL-21 6.5 pg/ml,
